# Long-Term Follow-Up of Nonsurgical Endodontic Treatments Performed by One Specialist: A Retrospective Cohort Study about Tooth Survival and Treatment Success

**DOI:** 10.1155/2020/8855612

**Published:** 2020-11-22

**Authors:** Paolo Mareschi, Silvio Taschieri, Stefano Corbella

**Affiliations:** ^1^Private Practice, Spilimbergo (UD), Italy; ^2^Department of Biomedical, Surgical and Dental Sciences, Università degli Studi di Milano, Milan, Italy; ^3^IRCCS Istituto Ortopedico Galeazzi, Milan, Italy; ^4^Institute of Dentistry, Department of Oral Surgery, I. M. Sechenov First Moscow State Medical University, Moscow, Russia

## Abstract

**Background:**

The main aim of the retrospective cohort study was to evaluate tooth survival after the endodontic treatment over a period of more than 20 years. Moreover, success of the treatment and the correlation between baseline parameters and the outcomes were analyzed, and causes were recorded.

**Materials and Methods:**

Clinical records (including radiographs) of subjects treated with endodontic procedures (both primary and secondary (nonsurgical retreatment)) were collected and analyzed, covering a period of up to 29 years. Type of the treatment, technique, adequacy of treatment performed, presence of baseline radiolucency, and symptoms at baseline were recorded. Moreover, failure (presence of radiolucency 2 years after treatment) and tooth extraction data and causes of them were recorded. Outcomes were explored by using survival analysis (Kaplan–Meier estimates and survival table analysis) and regression analysis (Cox regression).

**Results:**

A total of 2,679 endodontically treated teeth were included in the analysis. After 20 years from the treatment, the cumulative survival rate for primary and secondary treatments was 84.10% (80.99%–87.21%) and 89.79% (86.68%–92.90%), respectively. No differences were found between primary and secondary treatments or with regard to the technique adopted. The presence of periapical radiolucency was correlated to higher odds of tooth extraction.

**Conclusions:**

Despite the limitations of the study, we can assume that the proportion of retained endodontically treated teeth was significantly high over a long-term period.

## 1. Introduction

The aim of the nonsurgical endodontic treatment is the removal of pulp tissues, debris, and microbial agents and to create the conditions allowing the complete and effective tridimensional filling and sealing of the root canal system [1–3].

The success of the endodontic treatment was generally evaluated as the absence of apical periodontitis as it is detectable by clinical and radiographic investigation [4]. However, from the patients' point of view, the survival of treated teeth and their retention in function should be considered more reliable and more comparable to the outcomes used to evaluate the success of other dental treatments, such as implant and periodontal ones [5, 6]. In particular, tooth survival becomes an important factor to be considered when evaluating endodontic treatment outcomes over a long-term period [6].

One study conducted by Salehrabi and Rotstein, published in 2004, reported the results about teeth survival after mining an insurance database of about 1,462,936 treated teeth [7]. The authors reported that 97% of teeth were retained in the oral cavity after 8 years from the treatment, and they found that teeth without crowns were, in general, more prone to be lost than teeth covered with prosthetic crowns. With particular regard to nonsurgical orthograde retreatments, one paper reported the results of one study on 4,744 teeth followed up for a period of 5 years [8]. After such period, 89% of teeth were retained in the oral cavity functioning.

Furthermore, some authors, in a randomized controlled clinical trial, demonstrated that the endodontic treatment demonstrated the same success rates of dental implants, in the short term, even in teeth with uncertain prognosis [6, 9].

Among the causes of failure, Olcay and coworkers found that restorative/endodontic reasons were the most frequent, and the most common reason for extraction was problems related to prosthetic reconstruction [10]. The importance of type and size of postendodontic restoration was also explored in another study on molars [11]. The authors found that when teeth were not covered by one prosthetic crown, the crucial factor was represented by the amount of remaining coronal tooth structure. Pratt and colleagues in 2016 found that endodontically treated teeth restored with composite/amalgam restorations were 2.29 times more prone to be extracted than teeth restored with full crowns over an eight-year period [12].

Setzer and colleagues also found that preoperative conditions (periodontal status and clinical attachment loss) could have an influence in worsening the prognosis of endodontically treated teeth [13].

Considering tooth-related factors affecting long-term outcomes, Ricucci and coauthors, in 2011, found that more severe conditions (pulp necrosis, apical periodontitis, and periapical lesion size larger than 5 mm) negatively influenced the outcomes of the endodontic treatment [14].

The aim of the present study is to report long-term outcomes (up to 29 years) of endodontically treated teeth, focusing on survival rates and on factors influencing tooth retention in the oral cavity.

## 2. Materials and Methods

The subjects in the present study were treated following the principles enlisted in the Helsinki Declaration and its further modifications [15]. The present study is an observational retrospective cohort study performed on a cohort of patients treated by one operator (PM) in one single clinical center in Spilimbergo (UD), Italy. All patients provided informed consent for intervention before any procedure, and they were treated by the same operator in one single center in Italy in the period ranging from 1988 to 2017. Because of the use of only retrospective fully anonymized data, this study was a nonintervention clinical trial, adopting a standardized nonexperimental protocol, without the need for local review board approval according to the European Guidelines for Good Clinical Practice (CPMP/ICH/135/95).

The paper was written in accordance to “Strengthening the reporting of observational studies (STROBE)” recommendations.

### 2.1. Eligibility Criteria

To be included in the study, the clinical records must (i) belong to subjects aged more than 18 years at the time of intervention, able to understand, and sign a written consent form; (ii) be about primary or secondary nonsurgical root canal treatment (nonsurgical retreatment); (iii) be complete, containing information about preoperatory status, characteristics of the treatment, and about the clinical course; (iv) belong to subjects without any disease/condition affecting the immune system; and (vi) be cases not showing perforation, root resorption, root fractures, or endoperio lesions.

### 2.2. Treatment Procedures

Three treatment procedures were adopted by the same experienced operator (PM): i) from 1988 to 1997, technique 1 (T1); ii) from 1998 to 2007, technique 2 (T2); and iii) from 2008 to 2017, technique 3 (T3).

Depending on the tooth location, local or inferior alveolar block anesthesia was administered. All teeth were treated after isolating the field with the rubber dam.

Briefly, in T1, the root canals were scouted by using #08, #10, and #15 stainless steel (SS) files before preflaring with #1 and #2 Gates burs; working length (WL) was determined using periapical radiographs with #08; “step-down” technique was adopted using #15, #20, and #25 SS files, and a radiograph was taken to verify the WL; then, the “step-back” procedure was adopted using a sequence of SS files of growing diameter; the obturation was obtained by warm vertical gutta-percha compaction.

In T2, after scouting and preflaring, LightSpeed™ endodontic system (Kerr Corporation, Orange, CA, United States) was used; the canal was obturated using the continuous-wave condensation technique.

In T3, after scouting, the root canals were prepared by mechanical instruments (MtwoⓇ, Sweden & Martina SpA, Due Carrare (PD), Italy) using a standard technique; the canal was obturated using the continuous-wave condensation technique.

In all techniques, 5% sodium hypochlorite was used as a disinfectant alternated to the use of 10% EDTA (used only in T3). Definitive restoration was placed in all cases within one month from root canal obturation.

### 2.3. Outcomes and Data Extraction

The primary outcome was to evaluate the long-term (20 years or more) survival rate (CSR%) of endodontically treated teeth in a single private practice. The condition of survival was the presence of the tooth in the mouth.

The secondary outcomes were as follows: i) success rate as evaluated through clinical (absence of symptomatology after 1 month from intervention) and radiographic evaluation (significant healing of periapical lesion/absence of periapical lesion); one treatment was considered successful in the absence of any clinical and radiographic sign; ii) impact of characteristics of the treatment on survival and success rate; iii) impact of baseline variables (tooth characteristics and preoperative conditions) on outcomes of the treatment.

To respond to the research question, the authors extracted the following parameters from the electronic clinical records available: gender of the subject, age of the subject, tooth, technique used, treatment (primary or secondary), data of the treatment, presence of baseline radiolucency, presence of baseline symptomatology (spontaneous or provoked), number of visits to perform the treatment, persistence of symptoms (spontaneous or provoked by percussion test), presence of radiolucency after treatment (after 3 and 5 years from the treatment), subjective evaluation of the adequacy of the treatment, tooth extraction, causes leading to tooth extraction, data of eventual extraction, data of the first follow-up visit after treatment, and data of the last follow-up after treatment. Radiographic evaluation was performed by two operators (PM and ST) independently, and disagreements were resolved by discussion; to define one treatment as adequate, the following criteria must be met: i) radiographic filling of the canal being within 1 mm from the radiological apex; ii) absence of overfilling; and iii) tridimensional filling of the root canal space without voids.

The data collection and analysis were performed on October 2019.

### 2.4. Statistical Methods

The statistics was performed by one operator (SC) using dedicated software (IBM SPSS Statistics version 22, IBM, Armonk, NY, USA).

Descriptive statistics was performed providing mean and standard deviation for continuous variables; for categorical variables, contingency tables were created, showing frequencies that were transformed into percentage values.

Survival and success rates were calculated by means of life table analysis and Kaplan–Meyer analysis. Differences among techniques and treatments were computed. Cox regression analysis served to evaluate the impact of baseline characteristics on survival curves. Logistics regression models were used to evaluate the correlation between the presence of symptoms after the treatments and the outcome and between the subjective evaluation of the treatment and the outcome. The level of significance was set at *P* *=* 0.05.

## 3. Results

**Figure 1:**
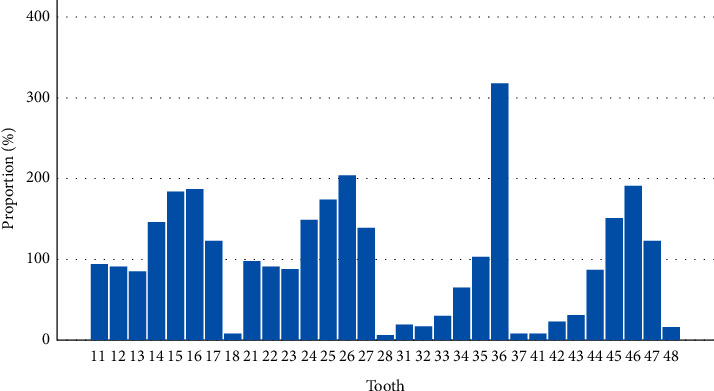
Proportions of treated teeth.

A total of 2,679 treatments in 1,097 subjects were included in the analysis, belonging to a population made of 59.4% of men (43.2 + − 14.6 years old). Eighty-four treatments (3.0%) were excluded mostly because of missing data at baseline (*n* = 74). Ten cases in subjects with diseases affecting the immune system were excluded. As for the techniques performed, Figure 1 shows the teeth distribution. T1 was adopted in 19.2% of teeth, T2 in 51.1% of teeth, and T3 in 29.7% of teeth; 66% of the total were primary treatments, while 33% were secondary ones.

With regard to the type of restoration, 30.5% of teeth were restored with indirect metal post and core and prosthetic crown, 10.0% with amalgam, 38.9% with direct composite, 7.6% with composite and post (carbon fiber), 9.2% with direct reinforced composite (Ti-CoreⓇ, Essential Dental Systems, Inc., New Jersey, United States), and 3.8% with direct reinforced composite (Ti-CoreⓇ) and carbon fiber post and prosthetic crown.

**Figure 2:**
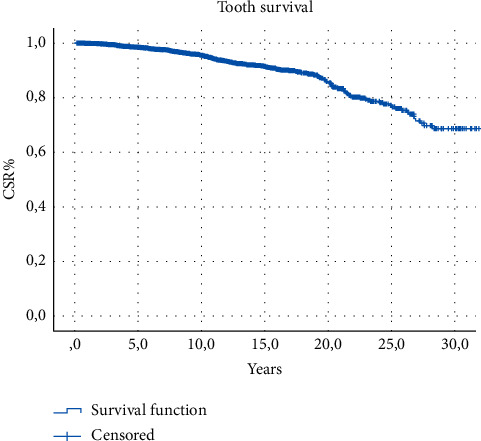
Survival function for all teeth (Kaplan–Meier).

**Figure 3:**
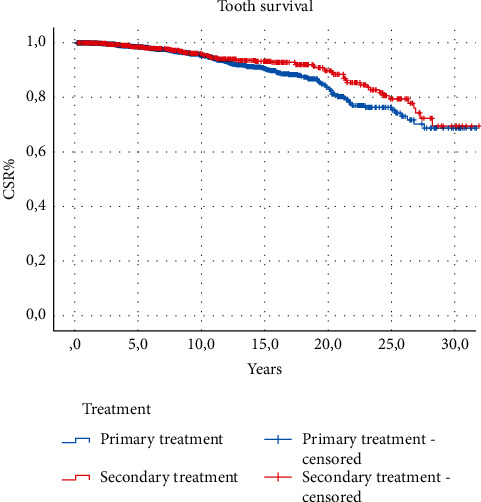
Survival function comparing primary and secondary treatments (Kaplan–Meier).

**Figure 4:**
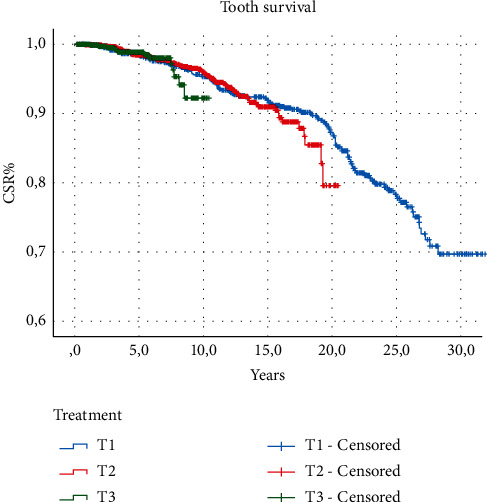
Survival function comparing different techniques (Kaplan–Meier).

The results of descriptive statistics of categorical variables are shown in Table 1. The cumulative survival proportions are represented in Table 2. The reported CSR% was 86.25% after 20 years from the treatment without statistically significant differences among the techniques adopted. Survival analysis did not show any significant difference between primary and secondary treatments and among the different techniques used (log-rank test, *P* > 0.05) regarding tooth survival (Figures 2–4). The results of the analysis of the influence of baseline conditions to teeth survival curves are shown in Table 3. Age, sex, and tooth location were not related to teeth survival rates. The presence of symptoms at the end of the treatment was not correlated to the outcome for both primary and secondary treatments. Among the causes of failure, the most frequent was the fracture of the tooth (50.5%); then, 21.3% were lost due to untreatable caries, 17.3% were lost due to periodontal reasons, and 10.9% were lost due to other reasons, including failure of the endodontic treatment.

The subjective evaluation of adequacy of the treatment was negatively correlated to the failure of the treatment (presence of periapical radiolucency) for both primary treatment (Exp(B) = 0.356, 95% CI: 0.167–0.761, *P* = 0.008) and retreatments (Exp(B) = 0.476, 95% CI: 0.281–0.806, *P* = 0.006). The presence of baseline radiolucency was correlated to the failure of the treatment (presence of periapical radiolucency) for both primary treatment (Exp(B) = 3.250, 95% CI: 1.852–5.701, *P* < 0.001) and retreatments (Exp(B) = 9.563, 95% CI: 4.101–22.304, *P* < 0.001). Technique T3 is positively correlated to better outcomes than T1 and T2 for primary and secondary treatments with the presence of radiolucency at baseline.

## 4. Discussion

The study reported long-term results after the endodontic treatment was performed in one single private practice setting.

To fully interpret the results, weighting their external validity, we have to acknowledge several limitations in the study design. Firstly, the retrospective design of the study could not be considered ideal to compare different techniques and treatments because a prospective design should be more adequate; moreover, primary and secondary endodontic treatments clearly found their application in clinical situations that are completely different, and this assumption must be considered an important limitation when evaluating the comparison made between the two treatments. The treatments in the study were all performed by the same experienced clinician; on the one side, this aspect provided an important factor to minimize the internal heterogeneity of data since the factors related to the operator, even considering his learning curve over time, can be considered homogeneous; on the other side, the results of the study could be extended to the general population with caution. Finally, the methods for defining the success of the endodontic treatment based on clinical (presence of signs and symptoms) and radiographic (evidence of periapical radiolucency) examination could be biased by the methods of visualization of the lesion [16–18].

The results of the study could be read in light of the existing literature.

In general, there is substantial evidence to affirm that the endodontic treatment is effective for obtaining tooth survival in medium- and long-term follow-up periods [6, 8, 13, 19–24]. The results obtained in the present research are substantially comparable to those reported by two authors who mined an insurance database in the United States in 2004 [8]. They reported that 97% of teeth treated (more than one million subjects included) were retained in the oral cavity in function after eight years from the endodontic treatment, while we reported a CSR% of 95.74% after ten years. The other papers reported the results of mining the insurance database, with relatively high survival rates, being 94% after 3.5 years of follow-up in the study conducted by Lazarsky and coworkers [20] and 93% after 5 years of observation in the study conducted by Chen and colleagues [25, 26] published in 2007 on a Taiwanese population. One recent study published in 2020 reported 79% of teeth that survived at the 20-year follow-up control, in a cohort of 130 patients (17% dropouts) treated by the same operator [26].

Another study published by Fonzar and coworkers in 2009, with a protocol similar to the one used in the present study, reported survival rates of a total of 1175 teeth [19]. Even though the reported CSR% after 10 years was provided without any measure of variability, we can assume that the 93% reported was not significantly different from what was found in our study. Interestingly, as it was also found in our results, the authors reported that even though survival rates were not significantly different between primary and secondary treatments, the proportion of teeth surviving after 10 years was higher in the retreatment group than in treatment one. This aspect should be deeply explored in further studies. As compared to the “practice-based” study published by Skupien and colleagues in 2013, who reported an annual failure rate of 1.88% [23], in our study, the proportion of teeth surviving after 10 years from the treatment is significantly higher.

Regarding the causes of teeth extraction, the majority of teeth lost in our cohort was extracted due to fractures. This consideration was supported by one paper on a large number of subjects treated with endodontic, periodontal, and prosthetic treatments which were followed up for a long period [27]. In addition, the study conducted by Lee et al., published in 2012, reported that root and crown fractures caused approximately half of the failures of the endodontic treatment [6]. However, such assumption appeared not to be supported by the results of the study conducted by Fonzar et al. that reported periodontitis as the most frequent cause of tooth extraction [19].

In our cohort, a radiolucency was detected in 2.5% of teeth that underwent primary endodontic treatment and in 5.6% of teeth that were retreated. Several studies were published presenting data about the success of the nonsurgical endodontic treatment as evaluated by using periapical radiographs and clinical examination [4, 6, 14, 22, 26, 28, 29]. The scientific literature reported results that were significantly heterogeneous, ranging from 31% to 96%, as reported in the systematic review of the literature published by Ng et al. in 2007 [30, 31]. However, some authors hypothesized that many limitations could be found in previously published systematic reviews of the literature exploring the outcome of the endodontic treatment, thus limiting the possibility to generalize the results to the entire population [32]. Moreover, some authors reported that changes in periapical radiolucency could be observed even after more than 20 years from the treatment [33]; thus, we can assume that the adoption of just radiographic parameters to evaluate the success of one endodontic procedure could underestimate the outcome of the treatment.

Amongst the factors affecting the survival of endodontically treated teeth, we found that the presence of baseline radiolucency was correlated to a higher risk of tooth loss, and this was reported to be more significant for the primary treatment. Such observation appeared to be in contrast to what was found in the systematic review published by Ng and coworkers in 2010 [24] and in the study conducted by the same study group on a cohort of 1,617 endodontically treated teeth [21]. However, the correlation between baseline radiolucency and tooth survival has been reported in other studies from different countries [6, 34, 35].

Regarding the success of the endodontic treatment, the presence of baseline radiolucency significantly reduced the odds of success for both primary and secondary treatments. Such result was consistent to what was found in other published studies [22, 31]. Moreover, as it was reported by our results, the adequacy of treatment (as evaluated by radiographic parameters immediately after treatment) influenced importantly the possibility to obtain the success of the treatment, in accordance to what was found in other studies [6, 14, 22].

Regardless of the limitations of the study which were described above, we can conclude the following:

- In this particular cohort of patients treated by one specialist in one private practice setting, tooth retention was very high even after a long-term follow-up period (86.25% after 20 years)
- Neither the technique nor the type of treatment (primary or secondary) appeared to affect the survival rate; the presence of baseline radiolucency was related to lower odds of tooth survival over the years
- The success of the treatment was correlated to the absence of baseline radiolucency and to the adequacy of the treatment performed

More prospective studies should assess not only short-term outcomes but also long-term ones to better understand factors affecting tooth survival and the success of the endodontic treatment.

## Figures and Tables

**Table 1:** Distribution of categorical variables.

		Treatment adequacy		Final symptomatology			Final radiolucency		Extraction
		Adequate	Inadequate	Absent	Provoked	Spontaneous	Absent	Present	
Treatment	Primary (*N* = 1779)	1657 (93.1%)	122 (6.9%)	1679 (94.4%)	90 (5.0%)	10 (0.6%)	1730 (97.2%)	49 (2.8%)	128 (7.2%)
	Secondary (*N* = 900)	593 (65.9%)	307 (34.1%)	871 (96.8%)	26 (2.9%)	3 (0.3%)	843 (93.7%)	57 (6.3%)	60 (6.7%)
Technique	T1 (*N* = 787)	658 (83.6%)	129 (16.4%)	761 (96.7%)	26 (3.3%)	0 (0.0%)	740 (94.0%)	47 (6.0%)	104 (13.2%)
	T2 (*N* = 1261)	1065 (84.5%)	196 (15.5%)	1224 (97.1%)	36 (2.8%)	1 (0.1%)	1226 (97.2%)	35 (2.8%)	74 (5.9%)
	T3 (*N* = 631)	527 (83.5%)	104 (16.5%)	565 (89.5%)	54 (8.6%)	12 (1.9%)	607 (96.2%)	24 (3.8%)	10 (1.6%)
Baseline radiolucency	Absent (*N* = 1912)	305 (15.9%)	1607 (84.1%)	1852 (96.9%)	53 (2.8%)	7 (0.3%)	1880 (98.3%)	32 (1.7%)	109 (5.7%)
	Present (*N* = 767)	124 (16.2%)	643 (83.8%)	698 (91.0%)	63 (8.2%)	6 (0.8%)	693 (90.4%)	74 (9.6%)	79 (10.3%)
Baseline symptomatology	Absent (*N* = 1611)	1280 (79.5%)	331(20.5%)	1590 (98.7%)	19 (1.2%)	2 (0.1%)	1564 (97.1%)	57 (3.5%)	114 (7.1%)
	Provoked (*N* = 619)	552 (89.2%)	67 (10.8%)	563 (91.0%)	53 (8.5%)	3 (0.5%)	588 (95.0%)	31 (5.0%)	40 (6.5%)
	Spontaneous (*N* = 439)	400 (91.1%)	39 (8.9%)	388 (88.4%)	43 (9.8%)	8 (1.8%)	421 (95.9%)	18 (4.1%)	25 (5.7%)

**Table 2:** Cumulative survival rate over time.

Time frame	Primary treatment			Secondary treatment			All		
	*n*	CSR%	95% CI	*n*	CSR%	95% CI	*n*	CSR%	95% CI
0–5 years	1779	98.45	97.85%–99.05%	900	98.62	97.83%–99.41%	2679	98.51	98.03%–98.99%
6–10 years	1286	95.82	94.73%–96.91%	683	96.08	94.62%–97.54%	1969	95.91	95.03%–96.79%
11–15 years	858	90.75	88.85%–92.65%	465	92.95	90.74%–95.16%	1323	91.53	90.07%–92.99%
16–20 years	430	84.10	80.99%–87.21%	261	89.79	86.68%–92.90%	691	86.25	83.98%–88.52%
21–25 years	175	75.39	70.19%–80.59%	144	81.40	75.79%–87.01%	319	77.72	73.88%–81.56%

**Table 3:** Influence of baseline parameters on teeth survival.

		Primary treatment			Secondary treatment			All		
		Exp (B)	95% CI Exp (B)	Sign	Exp (B)	95% CI Exp (B)	Sign	Exp (B)	95% CI Exp (B)	Sign (*p*)
Treatment	Primary	—	—	—	—	—	—	1.000 (1)	—	—
	Secondary	—	—	—	—	—	—	1.323	0.972–1.800	0.075
Technique	1	1.000 (1)	—	—	1.000 (1)	—	—	1.000 (1)	—	—
	2	1.138	0.755–1.714	0.536	1.174	0.611–2.256	0.631	1.129	0.847–1.697	0.307
	3	1.130	0.455–2.807	0.793	1.793	0.565–5.694	0.322	1.369	0.670–2.797	0.389
Baseline radiolucency	Absent	1.000 (1)	—	—	1.000 (1)	—	—	1.000 (1)	—	—
	Present	1.961	1.361–2.826	<0.001	1.551	0.927–2.595	0.095	1.607	1.201–2.149	0.001

## Data Availability

The clinical and radiographic data used to support the findings of this study have not been made available because of privacy regulamentations.

## Supplementary Materials

Supplementary Materials STROBE statement—checklist of items that should be included in reports of observational studies.
